# Population genomic analyses reveal high diversity, recombination and nosocomial transmission among *Candida glabrata* (*Nakaseomyces glabrata*) isolates causing invasive infections

**DOI:** 10.1099/mgen.0.001179

**Published:** 2024-01-16

**Authors:** Yue Wang, Jianping Xu, Fatma Ben Abid, Husam Salah, Sathyavathi Sundararaju, Khalil Al Ismail, Kun Wang, Lisa Sara Matthew, Saad Taj-Aldeen, Emad B. Ibrahim, Patrick Tang, Andres Perez-Lopez, Clement K. M. Tsui

**Affiliations:** ^1^​ Department of Biology, McMaster University, Hamilton, Ontario, Canada; ^2^​ Department of Medicine, Division of Infectious Diseases, Hamad Medical Corporation, Doha, Qatar; ^3^​ Weill Cornell Medicine-Qatar, Doha, Qatar; ^4^​ Communicable Disease Centre, Hamad Medical Corporation, Doha, Qatar; ^5^​ Division of Microbiology, Department of Laboratory Medicine and Pathology, Hamad Medical Corporation, Doha, Qatar; ^6^​ Division of Microbiology, Department of Pathology, Sidra Medicine, Doha, Qatar; ^7^​ Research Department, Sidra Medicine, Doha, Qatar; ^8^​ Infectious Diseases Research Laboratory, National Center for Infectious Diseases, Tan Tock Seng Hospital, Singapore, Singapore; ^9^​ Lee Kong Chian School of Medicine, Nanyang Technological University, Singapore, Singapore; ^10^​ Faculty of Medicine, University of British Columbia, Vancouver, BC, Canada

**Keywords:** antifungal susceptibility, candidaemia, comparative genomics, Middle East, nosocomial

## Abstract

*Candida glabrata* is a commensal yeast of the gastrointestinal tract and skin of humans. However, it causes opportunistic infections in immunocompromised patients, and is the second most common *Candida* pathogen causing bloodstream infections. Although there are many studies on the epidemiology of *C. glabrata* infections, the fine- and large-scale geographical nature of *C. glabrata* remain incompletely understood. Here we investigated both the fine- and large-scale population structure of *C. glabrata* through genome sequencing of 80 clinical isolates obtained from six tertiary hospitals in Qatar and by comparing with global collections. Our fine-scale analyses revealed high genetic diversity within the Qatari population of *C. glabrata* and identified signatures of recombination, inbreeding and clonal expansion within and between hospitals, including evidence for nosocomial transmission among coronavirus disease 2019 (COVID-19) patients. In addition to signatures of recombination at the population level, both MATa and MATα alleles were detected in most hospitals, indicating the potential for sexual reproduction in clinical environments. Comparisons with global samples showed that the Qatari *C. glabrata* population was very similar to those from other parts of the world, consistent with the significant role of recent anthropogenic activities in shaping its population structure. Genome-wide association studies identified both known and novel genomic variants associated with reduced susceptibilities to fluconazole, 5-flucytosine and echinocandins. Together, our genomic analyses revealed the diversity, transmission patterns and antifungal drug resistance mechanisms of *C. glabrata* in Qatar as well as the relationships between Qatari isolates and those from other parts of the world.

## Abbreviations

CV, cross-validation; ECV, epidemiological cutoff value; FG, four-gamete; GWAS, genome-wide assiciation studies; HAI, hospital-acquired infection; MIC, minimum inhibitory concentration; MLST, multi-locus sequence typing; MTL, Mating type loci; NWT, non-wild-type; PCA, principal components analysis; WGS, whole genome sequencing; WT, wild-type.

## Impact Statement

In 2022, the World Health Organization listed *Candida glabrata* (*Nakaseomyces glabrata*) as one of seven high-priority human fungal pathogens that pose significant public health threats. *C. glabrata* exhibits intrinsically lower susceptibility to fluconazole, and its bloodstream infections are prevalent and frequently reported from various countries worldwide. In this study, we investigated the population genomics, epidemiology and resistance mechanisms of *C. glabrata* strains from patients in Qatar’s tertiary hospitals between 2015 and 2021. Genomic data revealed diverse genetic profiles, and coexistence of both mating types in the populations, as well as evidence of recombination among the Qatari strains. Whole-genome sequencing analysis demonstrated nosocomial transmission and putative outbreak events within the healthcare facilities during the COVID-19 pandemics, and global circulation of major genotypes. Also, several known and novel SNPs were identified as associated with antifungal resistance through genome-wide association studies. Understanding its genetic variability, the epidemiology of *C. glabrata* invasive infections and mechanisms for antifungal resistance is important to identify local transmission routes and inform appropriate therapy and infection control strategies.

## Data Summary

The raw sequencing reads are available from the National Center for Biotechnology Information (NCBI) under the BioProject accession number PRJNA980988.

## Introduction

*Candida glabrata* (known as *Nakaseomyces glabrata*) is a common commensal yeast of the gastrointestinal tract in healthy people [1–6]. In recent decades, bloodstream and deep tissue infections associated with *C. glabrata* have notably increased, making this fungus the second or third most common cause of invasive yeast infections after *Candida albicans* and/or *Candida tropicalis* [1, 2, 7]. For example, *C. glabrata* accounts for approximately one-fifth of total *Candida* bloodstream infections in many countries (e.g. China, France, Germany, Netherlands, UK, Canada and USA) [8–12]. Other infections caused by *C. glabrata* range from mild mucosal and skin infections including oral thrush [13], cutaneous granuloma [14] and vulvovaginitis [15] to more severe invasive candidiasis such as soft tissue abscess [16] and spondylodiscitis infections in patients with immunosuppressive conditions such as diabetes mellitus, acute myeloid and lymphoblastic leukaemia, stem cell and solid organ transplantation, critically ill patients receiving intensive care, and those recently exposed to broad-spectrum antibiotics. Recently, the World Health Organization (WHO) published a fungal priority pathogens list, with *C. glabrata* classified as a ‘high-priority’ pathogen, due to its intrinsically reduced susceptibility to fluconazole and other azoles [17].

Many studies have investigated the epidemiology and antifungal susceptibilities among isolates and populations of *C. glabrata*. Several types of molecular tools have been used to analyse the epidemiology of human fungal pathogens, including *C. glabrata* (e.g. [18, 19]). However, since 2003, multilocus sequence typing (MLST) has been the main method for genotyping and for investigating the spatial and temporal patterns of genetic variation in *C. glabrata* [20–24]. These studies have revealed contrasting patterns, such as low genetic relatedness in isolates recovered from the same geographical region as well as highly similar genotypes among distant locations. Besides MLST, other potential genetic markers such as DNA sequence variation at *MSH2* [25] for isolate or cluster differentiation have been described. *MSH2* is one of the DNA mismatch repair genes and its mutants have shown influence on antifungal resistance in *C. albicans* [26] and *C. glabrata* [25]. In recent years, whole-genome sequencing (WGS) has been increasingly used for analysing the phylogenetic relationships among isolates and populations of various human fungal pathogens [27]. For example, WGS of 149 *C. glabrata* isolates from seven countries (Australia, Belgium, France, Germany, Italy, UK and USA) revealed evidence for recombination as well as hyper-variations within the mitochondrial genomes, virulence genes and antifungal drug targets [28]. This hypervariability was partially attributed to the diverse geographical and ecological sources of the studied isolates. Meanwhile, antifungal drug susceptibility surveys [12, 29–32] have revealed that the prevalence of resistance to fluconazole varied between 6.1 and 17 % among clinical isolates worldwide. Patients with invasive infections caused by drug-resistant *C. glabrata* usually showed poor prognosis [33, 34]. According to Won *et al*., 64 patients with fluconazole-resistant *C. glabrata* bloodstream infections had a 30 day mortality rate of 60.9 %, and the 90 day rate was around 78.2 % [35]. Reported genetic drivers of azole resistance in *C. glabrata* include upregulation of the efflux pump genes *CDR1* and *CDR2* [35]. In addition, mutations in genes encoding transcription factors, such as *PDR1*, *ADA2* (a transcription adaptor of the Spt–Ada–Gcn5 acetyltransferase complex), *UPC2* and *TAC1* have been linked to azole resistance in *C. glabrata* [36–40]. Similarly, mutations in *ERG6* and *ERG11*, two genes involved in ergosterol biosynthesis and the targets of azole drugs, have also been linked to azole resistance in *C. glabrata* [41, 42]. Aside from azoles, resistance to echinocandins has also been reported [43–45], and several molecular mechanisms have been identified, including specific mutations in hotspot regions of *FKS1* and *FKS2* [46]*,* which encode the subunits of β-1,3 glucan synthase, the target of echinocandins [47, 48].

*C. glabrata* is phylogenetically closer to *Saccharomyces cerevisiae* [49] than to most other *Candida* species and it harbours three mating type (MAT)-like loci (*MTL*); however, these three *MTL* loci in *C. glabrata* are located on two different chromosomes [50]. The *MTL* and *HML*-like cassette are located on chromosome B while the *HMR*-like cassette is located on chromosome E. The *MAT* locus encodes either the *MAT*a1 gene in mating type **a** strains, or the *MAT*α1 and *MAT*α2 genes in mating type **α** strains. The HML-like cassette contains homologues of *MAT*α1 and *MAT*α2 genes, while the HMR-like cassette has a homologue of the *MAT* **a** gene [51]. Mating type switching of *C. glabrata* has been demonstrated to occur in both *in vivo* and *in vitro* conditions. *MTL* **a** to *MAT* **α** switching was observed at the site of colonization [52], while switching could be induced in both directions under laboratory conditions, but this was accompanied by a high cell mortality [53].

The State of Qatar is located on the northeast coast of the Arabian Peninsula with a subtropical desert climate. We hypothesize that due to the unique environment, populations of *C. glabrata* in Qatar are likely to be different from those in other regions. On the other hand, since expatriates constitute more than 85 % of Qatar’s population, including a significant population from the Indian subcontinent [54], it has a high ethnic diversity and recent potential anthropogenic influences from other parts of the world. Specifically, in 2019, ~25 % of the Qatari population was estimated to be Indian nationals, ~12 % each belonged to Bangladesh, Nepal and Egypt, followed by smaller percentages from many other countries in Asia, Europe and Africa [55]. These travel and human migration events could potentially facilitate the introduction of *C. glabrata* from different regions to Qatar. The small geographical region, unique climate,and large migrant population make Qatar an ideal location from which to examine how historical and contemporary factors may influence local population genetic diversity and drug susceptibility of *C. glabrata*.

There have been two previous studies on *C. glabrata* populations in Qatar. The first was a retrospective analysis of *Candida* isolates from 187 patients with 201 episodes of candidaemia [56]. Among these, 38 episodes were due to *C. glabrata*. However, all isolates were identified using MALDI-TOF MS, and the genetic relatedness among these isolates was not investigated. The second study examined 15 Qatari strains as part of a global study of 53 clinical *C. glabrata* isolates from various geographical regions [24]. These 15 strains belonged to four sequence types: ST3, ST15, ST46, and ST7, based on MLST. However, the small number of strains in the second study was insufficient to address either fine- or large-scale population genetic variation of *C. glabrata* in Qatar.

In this study, we analysed 80 *C*. *glabrata* isolates recovered mostly from invasive infections from six hospitals and healthcare facilities to explore population diversity and transmission dynamics within Qatar. We investigated the signatures of recombination and assessed genomic signatures of antifungal resistance. Furthermore, we studied the genomic relatedness between Qatari isolates and those from other parts of the world in a larger scale comparison.

## Methods

### Sample collection, identification, antifungal susceptibility measurements and epidemiology

A total of 80 non-repetitive *C. glabrata* isolates were obtained (Table S1, available in the online version of this article). These isolates were retrieved from patients admitted to six tertiary hospitals, including Hazm Meberik Hospital (HMGH), Hamad General Hospital (HGH), Heart Hospital (HH), Rhumailah Long Term Facility (RH), Sidra Medicine (SM) and National Center for Cancer Care and Research (NCCCR) between 2015 and 2021. Of note, HMGH was one of the hospitals designated for patients with COVID-19 pneumonia particularly to those in need of admission to an intensive care unit (ICU). Isolates were recovered mainly from blood and body fluids, in addition to urine and genital areas. Isolates were cultured on Chromogenic *Candida* agar (Oxoid) at 37 °C for 5–7 days following the manufacturer’s recommendations. Species identities were confirmed using MALDI-TOF MS (Bruker Daltonics). All isolates were maintained on Sabouraud Dextrose Agar (SDA) at 4 °C before DNA extraction and antifungal drug susceptibility testing.

Minimum inhibitory concentrations (MICs) for amphotericin B (Amb), fluconazole (Fluc), itraconazole (Ita), voriconazole (Vor), posaconazole (Pos), flucytosine (Fc), anidulafungin (And), caspofungin (Casp) and micafungin (Mica) were measured using either Sensititre YeastOne (TREK Diagnostic Systems), Vitek2 (bioMérieux) or Etest (bioMérieux) at various hospital sites. Clinical and Laboratory Standards Institutes (CLSI) breakpoints were used to interpret MICs for Fluc, And, Casp and Mica [57]. In addition, CLSI epidemiological cutoff values (ECVs) were used for Vor, Ita and Amb to separate wild-type (WT) from non-wild-type (NWT) isolates [58]. There is no CLSI breakpoint or ECV for Fc. To ensure consistency of our testing with recommended protocols, quality control strains *C. parapsilosis* ATCC 22019 and *C. albicans* ATCC 90028 were tested using the same antifungal susceptibility testing methods and MICs were interpreted following the CLSI guidelines (CLSI M27M44S).

Also, cases/strains (from the same hospital) forming clusters in the phylogenomic tree were carefully inspected as putative outbreaks. Epidemiological data and clinical information such as date of patient admission were reviewed to determine if those isolates were from hospital-acquired infection (HAI) or community-acquired infection based on the National healthcare safety network (NHSN) definition published in January 2021. An infection was defined as HAI if the date of event of the NHSN site-specific infection criterion occurred on or after the third calendar day of admission to an inpatient location where day of admission was calendar day 1. If the infection was identified within 2 days before admission or the day of admission to an inpatient location (calendar day 1) or the calendar day 2 after admission, the infection was considered present on admission (POA).

### Whole genome sequencing

Genomic DNA was extracted using a MasterPure Yeast DNA purification kit (Lucigen), and quantified using a Qubit 2 fluorometer (ThermoFisher). Genomic DNA libraries were constructed with a Nextera XT library preparation kit (Illumina), and the libraries were sequenced on an Illumina NextSeq for 300 cycles (150 bp paired-end) at the Integrated Genomics Services Department, Sidra Medicine.

### Mapping, variant identification and phylogenomic analyses

Raw read qualities were evaluated using Fastqc v0.11.9 [59]. Raw reads were adaptor and quality trimmed using Trimmomatic v0.39 [60]. SNP analysis was performed using the Snippy v4.6.0 pipeline (https://github.com/tseemann/snippy). Specifically, reads were aligned to the reference genome CBS 138 (GCA_000002545.2 ASM254v2) using BWA v0.7.17 [61]. SNPs were called using FreeBayes v1.3.6 [62] and filtered based on a minimum coverage of 10 and a quality of 100. The core SNP alignments were generated by concatenating SNP sites present in all genomes and were used to infer approximately maximum-likelihood phylogenetic trees using FastTree v2 [63]. The trees were visualized using iTOL v5 [64]. The distribution of SNPs in *MSH2* among the 80 isolates was interpreted [65].

To analyse the potential genetic distinctiveness (or relatedness) of the Qatari isolates, these Qatari genomes were compared with 149 publicly available *C. glabrata* genomes from seven countries, including the UK (68), Belgium (6), France (7), Germany (1), Italy (2), USA (12), Australia (51) and unknown origins (2). These genome sequences and reads were downloaded from BioProjects PRJNA361477 (65), PRJNA480138 (22) and PRJNA669061 (28). However, seven samples (SRR12825216, SRR12825218, SRR12825233, SRR12825253, SRR8068027, SRR5239754 and SRR5239755) from these three BioProjects were excluded from our analyses because they lacked genotype calls in over 50 % of the SNP loci.

### Recombination and genetic cluster analyses

The four-gamete (FG) test was conducted to infer recombination events for the total population (80 isolates) and for the various subpopulations inferred from phylogenetic analysis within the total population. In detail, for each tested (sub)population, biallelic SNP loci with the minor alleles present in at least two isolates were pairwise compared for FG SNP pairs. The incompatibility rate was defined as the FG SNP pairs divided by the total compared SNP pairs.

To identify the probable number of genetic clusters within the Qatari population of *C. glabrata*, the multi-sample variant call format of the Qatari population was converted to ped, bed and map file formats with PLINK v2.0 [66]. The data were analysed using Bayesian inference of genetic structure as implemented in ADMIXTURE v1.3 [67], employing an unsupervised model and varying the number of clusters (*K*) from 1 to 30, with 200 bootstrap replicates per *K* value. We used the *K* value with the lowest cross-validation (CV) error estimate as the optimal number of clusters in the Qatari population.

### Mating type locus determination

To determine the MAT of the Qatari *C. glabrata* isolates, blastn searches were conducted on assembled contigs of individual isolates against two reference sequences from the *MAT*a strain BG2 and the *MAT*α strain CBS138. In detail, *de novo* assembly was performed for each isolate using ABySS v2.2.5 [4] with the following parameter ranges: k=60–120, kc=1–3 and SPAdes v3.15 with default settings [68]. Assembled contigs of individual isolates were searched against the *MAT* **a** and *MAT* **α** reference sequences from BG2 and CBS138 using BLAST+ v2.13 [69]. The *MAT* **α** reference sequence starts from NC_005968.1 : 112 831 to 118 575 covering *MAT*α1 (CAGL0B01243g; 432 bp), *MAT*α2 (CAGL0B01265g; 561 bp), and *BUD5* (CAGL0B01287g; 3744 bp). The *MAT* **a** reference sequence ranges from CP048231.1 : 114 776 to 116 133 covering *MAT*a1 (CAGL0E00341g; 523 bp) and an upstream gene *EMG1* (CAGL0B01232g; 690 bp).

Due to the similarity among the *MAT* locus and the other two mating type-like loci (*HML* and *HMR*), we blasted the *MAT*α reference sequence against chromosome B from both reference strains and blasted the *MAT*a reference sequence against chromosome E from both reference strains and chromosome B from CBS138 to determine the matching criteria for mating type identification. Isolates would be considered *MAT*α if the start point of its scaffold-matched regions are present in nt1–432 and the end point in nt1450–5745 of the *MAT* **α** reference sequence, while isolates would be considered *MAT*a if the starting point and the end point of the scaffolds fall into regions nt1–460, and nt833–1358 of the *MAT*a reference. Strains that do not match the *MAT*a or *MAT*α reference sequences will be considered as having an ambiguous (or unidentified) mating type. Also, to identify potential copy number variations and gene losses/gains, read depth at the three MTL loci was estimated for each isolate using SAMtools v1.13 [70].

### Population genomic analyses

Population structure of *C. glabrata* was investigated using principal component analysis (PCA). Eigenvectors and eigenvalues were calculated based on core genome SNPs using PLINK v2.0 [66]. A scatter plot with first and second principal components (PCs) was generated using matplotlib [71]. Random noise drawn from a normal distribution was introduced to PC1 and PC2 using numpy [72]. Genome-wide nucleotide diversity (π), Tajima’s D (TD) and fixation index (Fst) were calculated from 10 kb windows across the genome using jydu/vcftools haploid mode [73]. Specifically, π and TD values were calculated for the Qatari population, population of isolates from other countries and the total population. Fst was calculated between the population from Qatar and that from other countries.

### Molecular mechanisms associated with antifungal resistance

Mutations potentially linked to antifungal resistance were inferred using the following approaches. First, SNPs of 83 genes that encode putative glycosylphosphatidylinositol-modified proteins [74] and 20 loci involved in antifungal resistance and virulence (e.g. *ERG11, FKS1, CDR1* and *PDR1* genes; Table S2) were extracted from each VCF file. We screened the 103 candidate genes for potential antifungally related SNPs by identifying unique non-synonymous mutations in strains with high MICs against antifungal drugs or above the ECV. Gene Ontology enrichment analysis was conducted on these candidate genes to infer their potential roles in antifungal resistance using FungiFun [75].

To identify SNPs in the above genes that are potentially linked to azole resistance, strains were divided into two groups: the ‘higher’ MIC group included those with MICs above the CLSI breakpoint for Fluc (>32 µg ml^−1^) as well as NWT samples with relatively high MICs in other azoles (≥32 µg ml^−1^ in Ita, Pos and Vor), while the remaining ones belonged to the lower MIC group (fluconazole susceptible/dose-dependent). Overall, 17 strains were grouped in the higher MIC group and 61 in the lower MIC group; two strains (SCG1 and cvcan1) missing MICs in at least three of the four azole drugs were excluded from this analysis.

In addition to the targeted analyses above for SNPs in known azole resistance-related genes, genome wide association studies (GWAS) were conducted for nine antifungal drugs to identify susceptibility-related SNPs for individual drugs. The nine drugs included in this analysis were AmB, Fc, Fluc, Vor, Ita, Pos, Casp, And and Mica. GWAS were performed using FarmCPU [76] from the R package GAPIT v3 [77]. The dependent variable for individual analyses was the MIC for each drug, and the independent variables included all SNP loci. Furthermore, to account for population structure and minimize its impact on the analysis, the top three to eight principal components of the independent variables were included as covariates.

## Results

### Genomic diversity in the Qatar population of *C. glabrata*

**Fig. 1.**
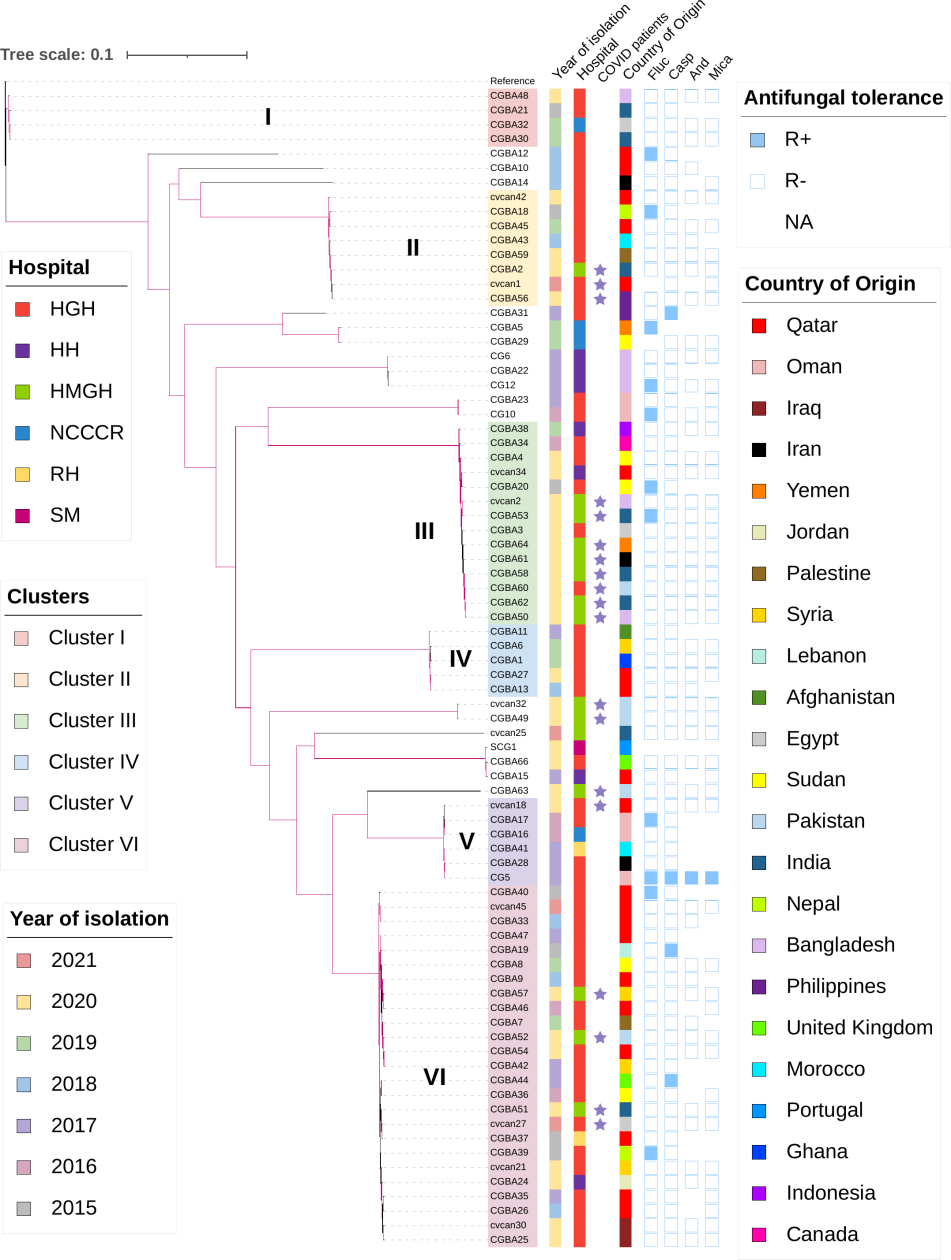
Genetic relationships of the 80 *Candida glabrata* isolates from Qatar inferred based on high-resolution SNPs generated by Snippy. Branches with bootstrap support >0.95 are highlighted in purple. The inner colour strips specify the year of isolation. The second inner colour strips specify the sources of the isolates (HMGH: Hazm Meberik Hospital, HGH: Hamad General Hospital, HH: Heart Hospital, RH: Rhumailah Hospital, SM: Sidra Medicine, NCCCR: National Center for Cancer Care and Research). Strains isolated from patients infected with COVID-19 were labelled with purple stars. The second inner colour strips indicate the county of origins of the patients. Clusters I–VI were highlighted with different colours. Resistance to fluconazole (Flu), caspofungin (Casp), anidulafungin (And) and micafungin (Mica) are indicated with blue squares. Blue squares represent resistance, while white squares indicate the absence of resistance. The tree scale measures evolutionary distance, corresponding to 0.1 substitutions per site (the denominator is the total number of SNPs).

In total, 80 *C*. *glabrata* isolates were sequenced and analysed in this study. These isolates were recovered from various body sites (74 from sterile sites) including blood (72; 90 %), ascites fluid (2; 2.5 %), urine (1; 1.25 %), genital swab (1; 1.25 %), abdomen fluid (2; 2.5 %), and pleural fluid (2; 2.5%) (see Table S1 for additional information). Based on mapping to the reference genome of strain CBS 138, we identified a total of 167 011 SNP sites in the Qatari population of *C. glabrata*, representing 1.4 % of the 12 Mb genome in this species. A high-resolution phylogenetic tree was generated to show the genomic relationships among the 80 Qatari isolates collected in six different hospitals (Fig. 1). The demographic characteristics of the host patients and the susceptibilities to four antifungal drugs for all 80 isolates are shown in Fig. 1. Among the 19 strains from COVID-19 patients, 14 were from HMGH and five were from HGH. These 19 strains showed a diversity of genotypes but with some of them from both hospitals clustering together. Similarly, many isolates were genetically similar to each other regardless of the nationality and the hospitals to which the patients were admitted. The number of SNPs between pairs of isolates in the Qatari population of *C. glabrata* ranged from 9 to 46 573 (Table S3). Phylogenomic analyses segregated the 80 isolates into different clusters based on their genetic divergence. In each of six tight clusters where four or more isolates were found in the Qatari population, the pairwise isolate SNP frequency was <0.004 % (Fig. 1, Table 1). Specifically, within each cluster, the average SNP difference between isolates was 451, 279, 194, 276, 231 and 254 respectively for clusters I, II, III, IV, V and VI (Table 1). In contrast, the average number of SNPs between isolates among different clusters were about 100× higher than those within these clusters (Table 1). After comparing the phylogenomic relationships among strains with epidemiological information (e.g. isolation years and hospitals) in individual clusters, we identified several putative clonal outbreaks as these strains were considered as HAI. For example, four cluster II isolates, CGBA56, CGBA59, cvcan42 and cvcan1, obtained from four patients between April 2020 and April 2021 from HGH, represented one probable outbreak cluster. Interestingly, this cluster also included three closely related strains, CGRA18, CGRA43 and CGRA45, collected in 2015, 2018 and 2019 respectively at HGH. Together, these results could indicate a persistent genotype cluster within HGH. Similarly, seven cluster III isolates from HMGH collected between May 2020 and September 2020, including four collected in June 2020, represented another putative clonal outbreak. Interestingly, those seven isolates were all collected from COVID-19 patients. In the same cluster (cluster III), three isolates, CGBA3, CGBA4 and CGBA60, were obtained from HGH between May and June 2020, suggesting that the outbreak may spread between the two hospitals. Evidence for outbreaks were similarly found for clusters IV and VI. In cluster VI, three strains, CGBA51, 52 and 57, were collected from HMGH between June and July 2020, with all from patients with COVID-19. Together, our results suggested persistent clonal strains of *C. glabrata* within and among hospitals in Qatar. In addition, there were 37 SNPs at the *MSH2* among the Qatari samples, and the unique SNP profiles in *MSH2* can be identified in each cluster (Fig. S1).

**Table 1.** Average SNP difference (and SNP difference in range) among *C. glabrata* isolates within and between the six clusters as defined in Fig. 1

Cluster	I	II	III	IV	V	VI
I	**451** (133–813)	33 169 (33 091–33 231)	44 934 (44 865–45 038)	39 890 (39 819–39 960)	40 274 (40 202–40 336)	34 874 (34 723–35 009)
II		**279** (26–523)	42 878 (42 817–43 011)	39 161 (39 110–39 226)	38 887 (38 859–38 924)	33 971 (33 830–34 100)
III			**194** (9–512)	43 682 (43 624–43 803)	43 593 (43 541–43 698)	38 424 (38 278–38 606)
IV				**276** (77–342)	37 568 (37 524–37 634)	32 734 (32 599–32 871)
V					**231** (45–334)	18 273 (18 139–18 405)
VI						**254** (37–421)

SNP: single nucleotide polymorphism.

### Mating type distribution and recombination

Among the 80 isolates, we were able to identify the mating type for 49 isolates, with the remaining 31 being ambiguous (or unidentified) (Table S1). Among the 49 isolates, 14 belonged to *MAT* **α**, 26 belonged to *MAT* **a** and nine appeared to contain both mating types. For the 31 isolates with ambiguous mating types, their MAT-bearing contigs were homologous to both the *MAT*a and *MAT*α reference sequences with over 98 % identity (Table S4). Interestingly, isolate CGBA20 did not have any contig that matched to the *MAT*a1 gene, which is consistent with the read depth findings for this isolate. Notably, among the six clusters of isolates, clusters II and VI contained both *MAT*a and *MAT*α isolates (Table S1), suggesting that recombination could occur among isolates within these clusters.

To investigate evidence for recombination in the Qatari population of *C. glabrata*, FG tests were conducted on the total Qatari population and the six individual clusters separately. Interestingly, the total population as well as five individual clusters all showed phylogenetic incompatibility, a common signature of recombination (Table 2). The only cluster without evidence of recombination was cluster I. However, there were only four isolates in cluster I, rendering detecting recombinant genotypes within this cluster extremely unlikely. For the remaining five clusters, the rate of SNP pairs showing evidence of recombination ranged from 25 to 46 %, with the highest rate in cluster III (Table 2). Given the low number of SNPs between pairs of isolates within each of the five clusters, the observed evidence for recombination probably reflected mating and genetic exchange between closely related strains and consistent with inbreeding within each of these clusters.

**Table 2.** Four-gamete tests on Qatari samples and individual subpopulations

Isolate cluster (no. of isolates)	No. of biallelic SNP loci with minor allele frequency > 2	No. of SNP pairs	No. of SNP pairs consistent with recombination (%)
In total (80)	136 178	9 272 155753	1 605 334 213 (17.31 %)
Cluster I (4)	176	15 400	0 (0 %)
Cluster II (8)	163	13 203	4015 (30.41 %)
Cluster III (14)	452	101 926	47 485 (46.59 %)
Cluster IV (5)	110	5995	1849 (30.84 %)
Cluster V (6)	240	28 680	7082 (24.69 %)
Cluster VI (25)	717	256 686	66 543 (25.92 %)

### Population structure

**Fig. 2.**
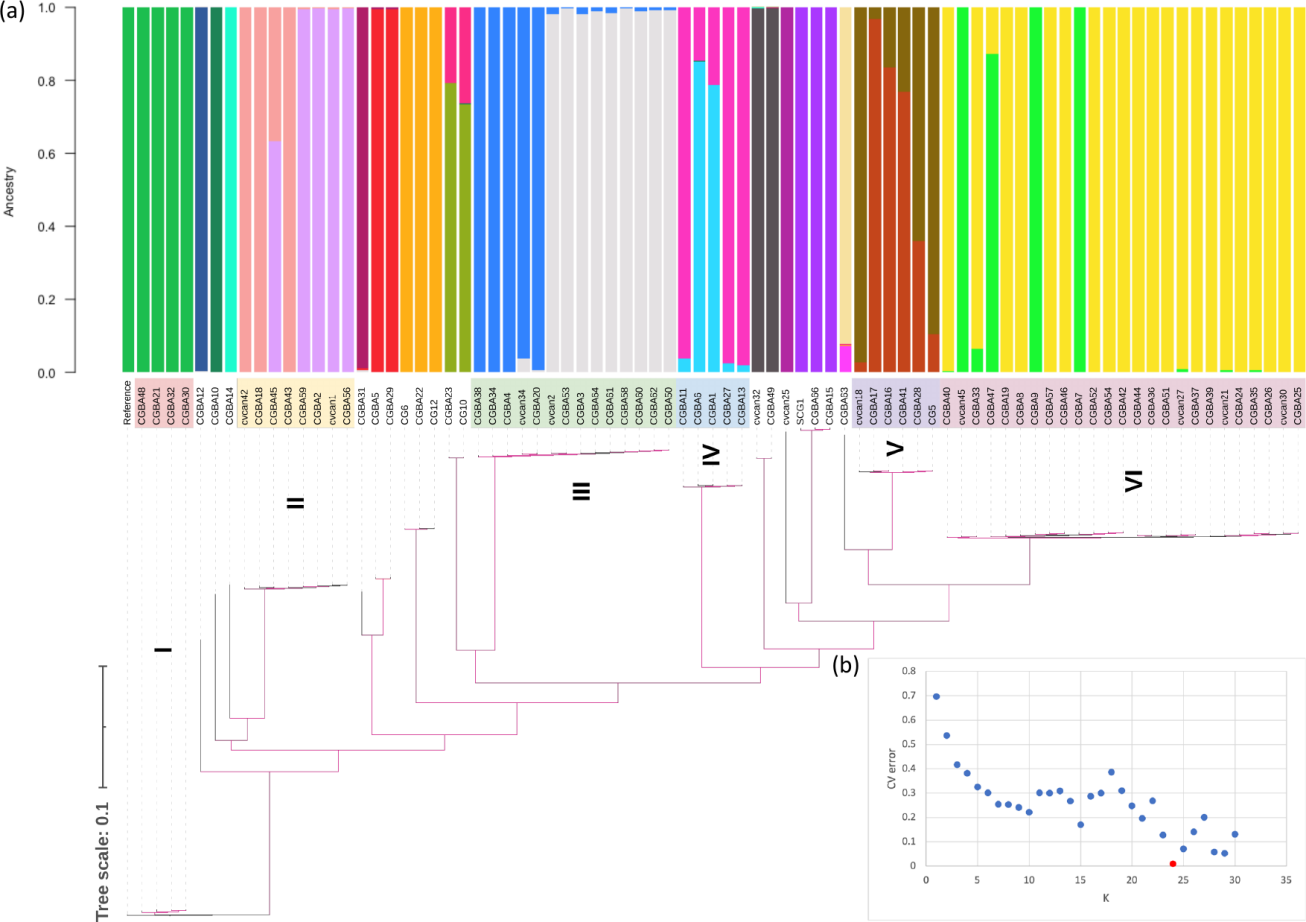
Ancestry and genetic admixture among individual and subpopulations of *Candida glabrata* populations. (**a**) Estimates of ancestry across the populations for Qatar isolates using *K *= 24 using ADMIXTURE analysis, revealing isolates from subpopulations (II–VI) with evidence of mixed ancestry. The ADMIXTURE plot is overlaid with the phylogenetic tree (Fig. 1). (**b**) The CV error from running unsupervised ADMIXTURE analysis, estimating *K* values from 1 to 30 with default settings and using a random seed. *K*=24 provided the lowest CV error.

In the above phylogenetic and recombination analyses, we focused on six clusters each containing at least four closely related isolates. To investigate the underlying genetic structure of the whole Qatari population of *C. glabrata*, we estimated the ancestry of all 80 isolates using ADMIXTURE (Fig. 2a). The estimated optimal ancestral population number (*K*) was 24 when CV error was the lowest (Fig. 2b). In the ancestral groupings of all 80 Qatari isolates, interestingly, except cluster I, all other five clusters contained isolates with mixed ancestries (Fig. 2a). Also, all six clusters as well as other sporadic isolates not belonging to the original six clusters contained genetic elements with unique ancestries (Fig. 2a). The evidence for multiple ancestries in many isolates was consistent with the hypothesis of recombination and outcrossing among isolates in different clusters.

### Antifungal susceptibilities and potential mechanisms of drug resistance

**Fig. 3.**
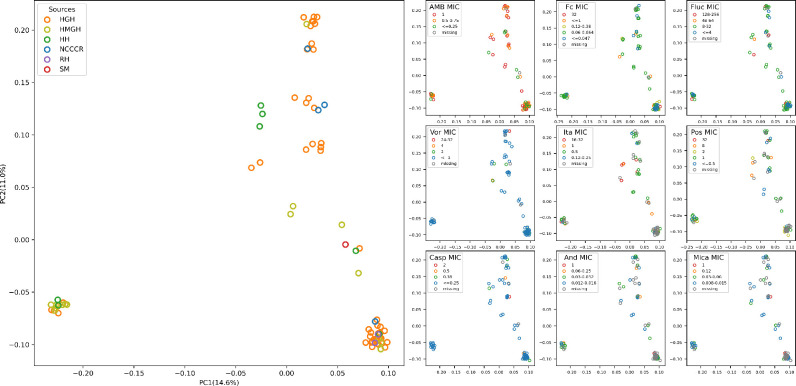
PCA of *C. glabrata* populations using high-resolution SNPs. PCA plot on the left shows the relationship of sampling source (hospitals), and the 25.6 % (14.6 %+11 %) genomic variance revealing little evidence of sub-clustering among isolation sources. Plots on the right-hand side labelled samples with different colours indicating different susceptibility against each of the nine tested antifungals. AMB: amphotericin B, Fc: flucytosine, Fluc: fluconazole, Vor: voriconazole, Ita: itraconazole, Pos: posaconazole, Casp: caspofungin, And: anidulafungin, Mica: micafungin.

The susceptibility data for all nine antifungals are summarized in Table S1. Overall, in the Qatari *C. glabrata* population, the prevalence of fluconazole resistance was 10 % while the echinocandin (Casp, And or Mica) resistance rate was 5 % (4/80). Only one isolate (CG5) was resistant to all three echinocandins. With respect to antifungal drugs without CLSI breakpoints, such as Fc, AmB, Ita, Pos and Vor, we found one, zero, seven, 44 and 68 isolates with their MICs greater than or equal to their ECVs. The NWT rates of *C. glabrata* for Ita, Pos and Vor ranged between 8.75 and 85 %. Three samples were resistant/NWT to all four azoles. To visualize the genetic relationships among isolates (Fig. S2) and how their clustering pattern might be related to antifungal susceptibilities, we plotted isolates onto a two-dimensional space using the top two principal components of their whole-genome SNPs (Fig. 3). Our data revealed broad distributions of isolates from different hospitals and with different drug-susceptibility patterns. In addition, most isolates with higher MICs (17 isolates) had high genetic similarities to isolates with low MICs (63 isolates), with the mean Fst estimate of 0.0007301 between the high- and low-MIC subpopulations. Together, these results were inconsistent with clonal expansion and dispersal of drug-resistant/tolerant strains in Qatar. Instead, isolates with high MICs probably originated independently multiple times from drug-susceptible ones and/or the drug resistance genes may be spread via sexual reproduction within and among populations (Fig. 3).

*C. glabrata* has intrinsically reduced susceptibility to fluconazole [78]. We tested whether mutations in any of the known antifungal or virulence-related genes were associated with the observed drug insensitivity. In total, 103 antifungal or virulence-related genes were screened for SNPs that caused missense mutations among the strains with higher (elevated) MICs. Candidate genes and their SNPs in the 80 Qatari *C. glabrata* sample are listed in Tables S2 and S5. Nineteen genes with missense mutations were identified exclusively present in the higher MIC group (Table 3). Furthermore, GO enrichment analysis suggested those genes played diverse biological functions. Fifteen GO terms were enriched (Table 4), including cell wall organization, hydrolase activity, adhesion of symbiont to host, hydrolase activity, among others. Additionally, four and one missense mutations in *PDR1* and *CDR2* were found prevalent within the higher MIC group, respectively, and each were identified in at least 14 of these 17 strains (Table 5).

**Table 3.** List of variants/missense mutations in genes that are associated with antifungal drug resistance or virulence in drug-tolerant isolates of *C. glabrata* in Qatar

Strain	Gene ID	Mutations	Annotation
CGBA21	*CAGL0K00110g*	c.1646A>T_p.Gln549Leu	Adhesive proteins
CGBA21	*CAGL0M08756g*	c.488G>A_p.Ser163Asn	Glycoside hydrolases II
CGBA12	*CDR1*	c.1333G>A_p.Asp445Asn	Multidrug transporter of ATP-binding cassette superfamily, involved in resistance to azoles
CGBA12	*CDR2*	c.82C>T_p.Arg28Cys	Multidrug transporter, involved in fluconazole resistance
CGBA12	*CAGL0I00220g*	c.322G>A_p.Asp108Asn	Adhesive proteins
CGBA12	*CAGL0I10340g*	c.998C>A_p.Thr333Lys	Adhesive proteins
CGBA12	*CAGL0I10340g*	c.1067G>A_p.Gly356Glu	Adhesive proteins
CGBA12	*CAGL0I10340g*	c.1609G>A_p.Asp537Asn	Adhesive proteins
CGBA12	*CAGL0G04125g*	c.434A>C_p.Gln145Pro	Adhesive proteins
CGBA12	*CAGL0J02508g*	c.2236G>A_p.Gly746Ser	Adhesive proteins
CGBA12	*CAGL0K13024g*	c.1121C>G_p.Thr374Arg	Adhesive proteins
CGBA12	*CAGL0K13024g*	c.1747T>C_p.Tyr583His	Adhesive proteins
CGBA12	*CAGL0K13024g*	c.2854G>A_p.Val952Ile	Adhesive proteins
CGBA12	*CAGL0E01595g*	c.1267C>T_p.Pro423Ser	Glycoside hydrolases II
CGBA12	*CAGL0E01595g*	c.1387G>A_p.Val463Ile	Glycoside hydrolases II
CGBA12	*CAGL0C02211g*	c.1409C>G_p.Ala470Gly	Glycoside hydrolases II
CGBA12	*CAGL0E01419g*	c.50C>T_p.Ala17Val	Partial aspartic protease
CGBA31	*SNQ2*	c.1466C>T_p.Ser489Phe	Involved in Pdr1p-mediated azole resistance
CGBA31	*CAGL0I00220g*	c.397A>C_p.Asn133His	Adhesive proteins
CGBA31	*CAGL0K13024g*	c.370A>G_p.Asn124Asp	Adhesive proteins
CGBA31	*CAGL0K13024g*	c.1184C>T_p.Ser395Leu	Adhesive proteins
CGBA31	*CAGL0K13024g*	c.1585G>A_p.Asp529Asn	Adhesive proteins
CGBA31	*CAGL0K13024g*	c.1589A>T_p.Lys530Ile	Adhesive proteins
CGBA31	*CAGL0M03773g*	c.428C>T_p.Thr143Ile	Unknown
CGBA20	*PDR1*	c.1744G>C_p.Val582Leu	Zinc finger transcription factor; regulates drug efflux pumps and controls multi-drug resistance
CGBA53	*PDR1*	c.1661A>G_p.Asp554Gly	Zinc finger transcription factor; regulates drug efflux pumps and controls multi-drug resistance
CGBA11	*CAGL0K00110g*	c.547G>C_p.Val183Leu	Adhesive proteins
CGBA5	*ERG6*	c.514G>T_p.Asp172Tyr	Mutation confers resistance to amphotericin B and cell-wall-affecting drugs
CGBA5	*CAGL0E01793g*	c.1267A>G_p.Ile423Val	Aspartic proteases
CGBA5	*CAGL0A03608g*	c.515C>T_p.Pro172Leu	Unknown
CGBA40	*CAGL0M04191g*	c.1663A>T_p.Asn555Tyr	Aspartic proteases

**Table 4.** Summary of the GO term enrichment for genes with non-synonymous mutations exclusively present in the high-MIC group

GO ID	GO name	Gene ID(s)	Exact *P*-value	Adjusted *P*-value	No. of genes/category	No. of genes/input
GO:0009277	Fungal-type cell wall	CAGL0C02211g | CAGL0J02508g | CAGL0K00110g | CAGL0K13024g	5.98014e-7	1.07643E-05	4/27	4/18
GO:0004190	Aspartic-type endopeptidase activity	CAGL0E01419g | CAGL0E01793g | CAGL0M04191g	9.08845E-06	8.1796E-05	3/16	3/18
GO:0005975	Carbohydrate metabolic process	CAGL0C02211g | CAGL0E01595g | CAGL0M08756g	0.000543456	0.002818744	3/61	3/18
GO:0016798	Hydrolase activity, acting on glycosyl bonds	CAGL0C02211g | CAGL0M08756g	0.000939581	0.002818744	2/17	2/18
GO:0004553	Hydrolase activity, hydrolysing O-glycosyl compounds	CAGL0C02211g | CAGL0M08756g	0.000939581	0.002818744	2/17	2/18
GO:0005618	Cell wall	CAGL0C02211g | CAGL0M03773g	0.000939581	0.002818744	2/17	2/18
GO:0006508	Proteolysis	CAGL0E01419g | CAGL0E01793g | CAGL0M04191g	0.00210127	0.005403266	3/97	3/18
GO:0005575	Cellular component	CAGL0A03608g | CAGL0E01793g | CAGL0I00220g | CAGL0I10340g | CAGL0K00110g | CAGL0M04191g	0.007741372	0.01741809	6/710	6/18
GO:0044406	Adhesion of symbiont to host	CAGL0K13024g	0.01102853	0.0219207	1/4	1/18
GO:0008233	Peptidase activity	CAGL0E01419g | CAGL0M04191g	0.01217817	0.0219207	2/62	2/18
GO:0000128	Flocculation	CAGL0I00220g	0.01376806	0.02252955	1/5	1/18
GO:0005199	Structural constituent of cell wall	CAGL0M03773g	0.0300587	0.04508805	1/11	1/18
GO:0031505	Fungal-type cell wall organization	CAGL0M04191g	0.03811053	0.04893688	1/14	1/18
GO:0016787	Hydrolase activity	CAGL0E01419g | CAGL0M04191g | CAGL0M08756g	0.03873213	0.04893688	3/281	3/18
GO:0071555	Cell wall organization	CAGL0C02211g	0.04078074	0.04893688	1/15	1/18

**Table 5.** List of common mutations associated with antifungal drug resistance in the higher MIC group

Gene	ID	POS	REF	ALT	No. of REF	No. of ALT	HGVS.c	HGVS.p
*CDR2*	CAGL0F02717g	NC_006029::263 877	A	T	1	16	c.2517A>T	p.Glu839Asp
*PDR1*	CAGL0A00451g	NC_005967::47 827	G	A	1	16	c.271G>A	p.Val91Ile
*PDR1*	CAGL0A00451g	NC_005967::47 849	T	C	1	16	c.293T>C	p.Leu98Ser
*PDR1*	CAGL0A00451g	NC_005967::47 782	T	C	3	14	c.226T>C	p.Ser76Pro
*PDR1*	CAGL0A00451g	NC_005967::47 983	A	C	3	14	c.427A>C	p.Thr143Pro

ID: gene ID; POS: SNP position; REF: reference allele; ALT: alternative allele; HGVS.c: Human Genome Variation Society – coding, represents the notation for describing DNA-level variations; HGVS.p: Human Genome Variation Society – protein, represents the notation for describing protein-level variations; No. of REF: counts of samples with reference allele at this locus; No. of ALT: counts of samples with alternative allele at this locus.

**Fig. 4.**
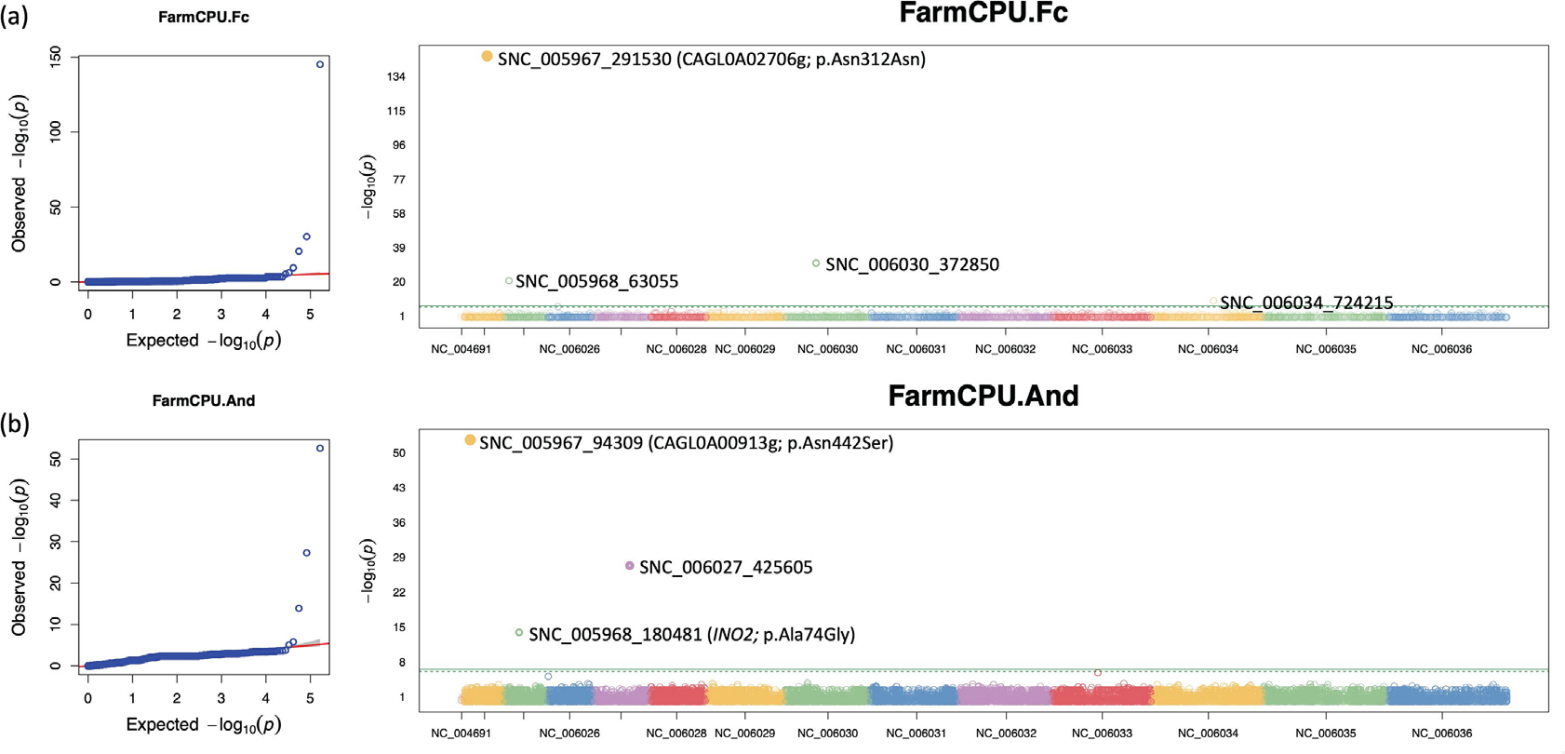
Q-Q plots and Manhattan plots of genome-wide SNPs associated with drug susceptibility to flucytosine (**a**) and anidulafungin (**b**). On the left, the *y*-axis of the Q-Q plot represents the quantiles of the observed *P*-values, while the *x*-axis represents the quantiles of the expected *P*-values under the null hypothesis. The red diagonal reference line represents the expected distribution of data. The blue circles deviating from the diagonal at the tail indicate that certain genetic variants are associated with drug susceptibility. On the right, each SNP was represented by a dot on the Manhattan plots. The *x*-axis shows the chromosomal positions of the markers. The *y*-axis represents −log_10_(*P*-value) for each SNP. Horizontal lines indicate the significance threshold to identify statistically significant associations for each analysis. Significant SNPs were labelled on the Manhattan plots and those coding region SNPs were annotated with gene ID and HGVS.p.

GWAS were conducted to further identify potential SNPs associated with variations in nine antifungal susceptibilities. Our analyses identified four SNPs and three SNPs associated with susceptibilities to flucytosine and anidulafungin, respectively (Fig. 4, Tables S6 and S7). For the four SNPs related to 5-flucytosine susceptibility, one synonymous mutation (Asn312Asn) was reported in CAGL0A02706g (Table S6), which was annotated to have a role in DNA damage checkpoint signalling and DNA repair [79]. The other three SNPs were in intergenic regions (Fig. 4). Regarding susceptibility to anidulafungin, GWAS identified three significantly associated SNPs (two were missense mutationss and the other was an intergenic SNP) (Table S7). One missense mutation converted asparagine to serine at position 442 in the protein product of CAGL0A00913g. The orthologue of CAGL0A00913g is known to be involved in cellular response to DNA damage, double-strand break repair and recombinational repair [79]. The other missense mutation resulted in a conversion of alanine to glycine at position 74 in the protein product of *INO2*, which encodes a transcriptional factor for the biosynthesis of inositol, a potential drug target for fungal infections [80, 81]. All the identified SNPs and detailed information are listed in Tables S6 and S7.

### Genomic comparison between samples from Qatar and other countries

To investigate the genetic relationships between Qatari isolates and those from other parts of the world, we retrieved the raw sequence reads from 149 global isolates. SNPs from all isolates were identified through mapping to the *C. glabrata* reference genome of strain CBS138, following the same procedures as described for the Qatari isolates. The SNPs were filtered, and unambiguous SNPs from all 229 isolates were retained for comparison. The filtered dataset included a total 10 429 SNPs among the 229 isolates.

**Fig. 5.**
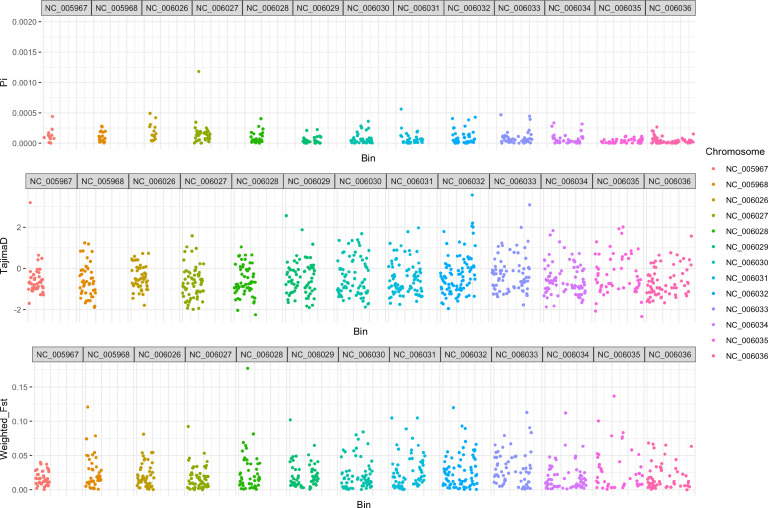
Population structure and genetic differentiation in *C. glabrata*. The π values and Tajima’s D were estimated for the total population (Qatar and other countries) based on 10 kb windows using the CBS138 reference genome. SNPs were masked if they were located in high-copy number (≥3) regions for the calculation of π values. The genome-wide pairwise Fst values between Qatar strains versus other strains were estimated based on 10 kb windows. The *x*-axis represents the sliding windows with scaffolds labelled on the top, and the *y*-axis shows the values for each estimate.

Nucleotide diversity analysis revealed that both the Qatar population and the population from outside of Qatar exhibited similar distributions of π, with mean values of 1.57×10^−4^ and 1.71×10^−4^ respectively. These values closely resemble the nucleotide diversity of the total sample, which was calculated at 1.64×10^−4^ (Fig. S3). Mean Tajima’s D values for the total, Qatar and outside of Qatar populations of *C. glabrata* were all negative, −0.44, –0.16 and −0.26 each (Fig. S3), consistent with recently expanding populations of this species. The high similarity between populations from Qatar and other parts of the worlds was further demonstrated by the low genome-wide Fst values across all scaffolds based on 10 kb windows (with a mean Fst value of <0.01) (Fig. 5). Interestingly, one genomic region, window NC_006028::280001–290 000, showed a high Fst value of 0.18. This region included seven putative genes: six protein-coding genes and one tRNA gene. Orthologues of these six genes have been identified to play roles in mitochondrial RNA processing, rRNA metabolic processing, RNA splicing, GCA codon-amino acid adaptor activity and N-terminal peptidyl-methionine acetylation.

**Fig. 6.**
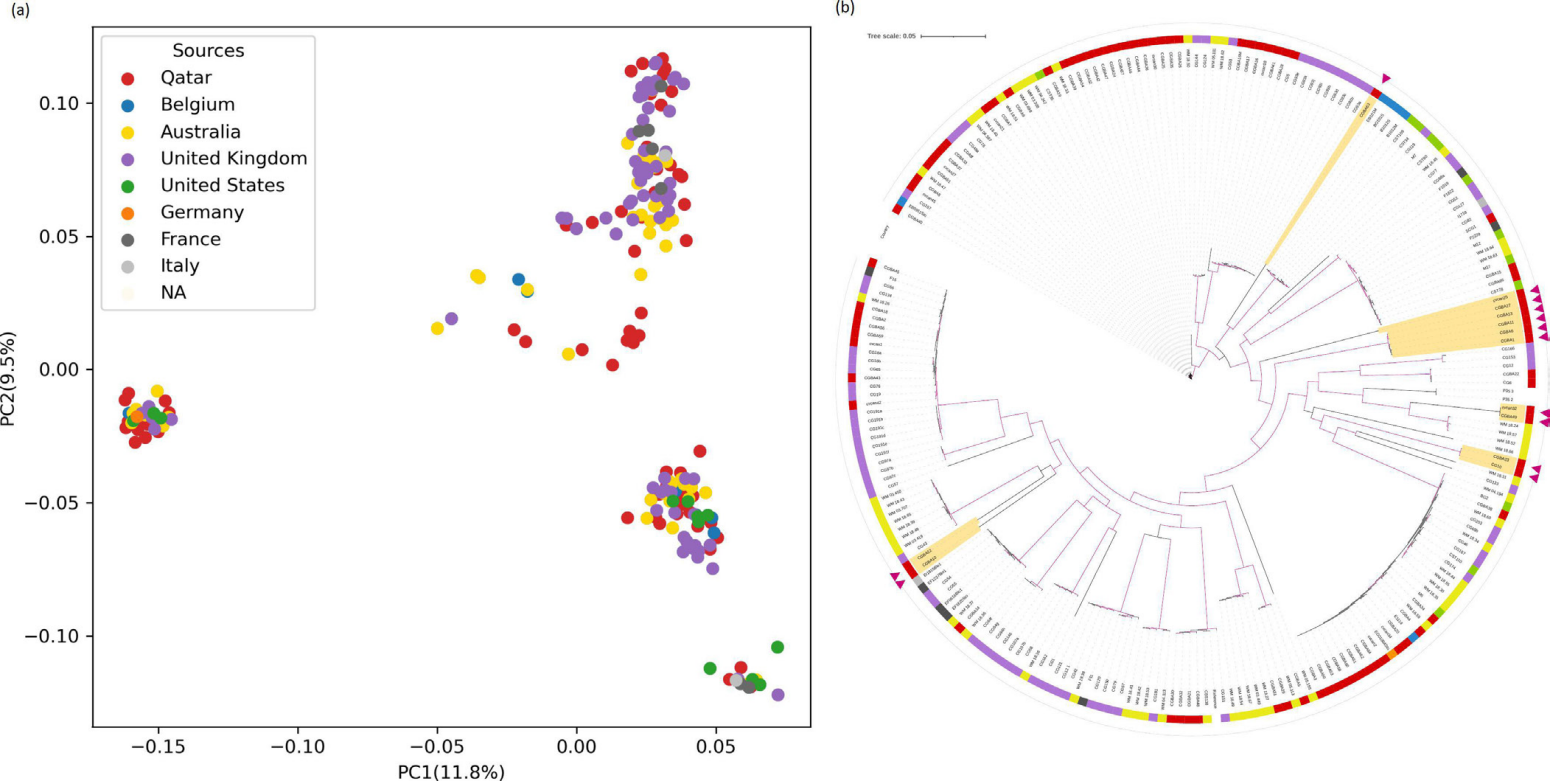
Genomic comparison between samples from Qatar and other countries based on high-resolution SNPs. (**a**) Clusters of 229 samples based on the first two principal components. (**b**) Genetic relationships of 80 Qatar isolates and 149 strains from other countries inferred based on core genome SNP alignments. Branches with bootstrap support >0.95 are highlighted in purple. The colour strip indicates the sources (country) of the isolates. Qatari strains with unique genotypes from the global strains are highlighted in yellow colour and marked with magenta triangles.

Consistent with the genome-wide Fst data, both the PCA plot and phylogenetic tree revealed that the Qatari isolates intermixed with isolates from the global collection. PCA illustrated similar genomic variation patterns between samples from Qatar and other countries, with the first two PCs accounting for 21.3 % of the total variation (Fig. 6a). However, eight Qatari isolates and those in cluster IV were divergent from isolates of the global collection from other parts of the world. These isolates including CGBA63, cvcan25, CG10, CGBA23, CGBA10, CGBA12, cvcan32, CGBA49, plus the five isolates CGBA27, CGBA13, CGBA1, CGBA6 and CGBA11 in cluster IV probably contained genomic variations unique to Qatar (Fig. 6a, b). Among the 10 429 SNPs, 1422 were exclusively present in these 13 isolates. They included 540 synonymous variants, 422 non-synonymous variants and 460 in non-coding regions (Table S8). The coding region SNPs are distributed among 548 genes. Each gene harboured from one to 14 SNPs, except for the gene CAGL0E06644g (*EPA1*), which contains 50 SNPs.

## Discussion

In this study, we analysed 80 *C*. *glabrata* isolates from six hospitals, collected from 2015 to 2021 in Qatar. Most isolates were recovered from candidaemia episodes, including 19 samples from COVID-19 patients. The genomes of all isolates were sequenced and analysed to identify the genetic relationships among isolates from the same and different hospitals. Our analyses revealed high genetic diversity in the Qatari *C. glabrata* population and evidence of nosocomial transmissions or even potential outbreaks among patients from within the same hospital and between hospitals. Among the 80 isolates, 19 were collected from patients reported to be positive for COVID-19, and many of them showed close genomic relationships with each other (Fig. 1). It is possible that prolonged hospital stay could have favoured the exposure and spread of *C. glabrata* among COVID-19 patients during the pandemics. Candidiasis outbreaks during the pandemics have been reported previously, mainly due to *C. albicans* and *C. auris* [82].

When compared to strains from other parts of the world, 13 Qatari strains had unique genotypes (Fig. 6). Interestingly, among these 13 strains, up to 50 SNPs were found in the gene *EPA1*, which encodes adhesin with a role in cell adhesion, and its upregulation has been reported to result in enhanced host colonization [83]. Stefanini *et al*. [27] suggested that *C. glabrata* may acquire resistance to antifungals by forming stronger aggregates between each other or adhering better to the host tissue. However, most strains from Qatar were intermixed with strains from other parts of the world, a result consistent with recent dispersal of *C. glabrata* around the world [27].

Evidence for both recent short- and long-distance dispersals has been reported for *C. glabrata* and several other human fungal pathogens, including *Aspergillus fumigatus* [84, 85]*, Candida auris* [86], *Candida albicans* [87], *Candida tropicalis* [88] and multiple lineages within the human pathogenic *Cryptococcus*, including in both the *Cryptococcus neoformans* and *Cryptococcus gattii* species complexes [89, 90]. However, in most of these molecular epidemiological and population genetic studies of human fungal pathogens, few isolates were from the Middle East region, an ecologically distinct region with a hot climate. In a previous study of *C. glabrata* that included isolates from Qatar, only 15 isolates belonging to four multilocus sequence types were analysed [24], making it difficult to assess the broad population genetic patterns within this species among different regions. Here, based on a large sample size, our analyses revealed that the strains from different sources (geographical regions and host ethnic groups) are mixed, consistent with recent migration of historically divergent clades. Human activities such as travel and trade between regions and countries are probably significant contributors to the observed dispersals.

Despite evidence for frequent dispersals among hospitals and regions, multiple genetically divergent isolates and clusters of *C. glabrata* were reported in Qatar. The patterns suggest the presence of unique and indigenous genetic diversity within Qatar, and that sexual mating and recombination among strains from divergent genetic clusters is not common. The presence of divergent lineages within the same geographical regions have been reported for several human fungal pathogens, including *C. auris* [86] and the human pathogenic *Cryptococcus* species complexes [89, 90]. In addition, the presence of divergent strains and clusters in both the Qatar and the global samples suggests historical genetic differentiations among subpopulations of *C. glabrata*. At present, whether the differentiated strains and genetic clusters were derived due to geographical or ecological isolation remains unknown. However, the presence of such clusters is indicative of cryptic speciation within the global population. Indeed, cryptic speciation has been reported for several human fungal pathogens including *C. albicans* [91, 92], *Aspergillus fumigatus* [93] and *Paracoccidioides brasiliensis* [93]. Cryptic fungal pathogens may pose significant identification and management challenges due to their morphological resemblance to known genotypes/species while harbouring unique genetic, antifungal and infection-relevant traits [94].

ADMIXTURE analysis estimated that 24 ancestral populations probably existed in the Qatari samples and about half of the isolates showed mixed ancestry (Fig. 2). Of note, there were fluctuations in CV errors around the optimal *k* value of 24. The observed fluctuations were probably due to complex genetic structure in the population where both clonal reproduction (including mutation accumulation and selection) and sexual reproduction existed. Sexual mating between divergent strains, even occasionally, can generate hybrids/recombinants and make it difficult to estimate the number of genetic clusters. Indeed, hybrids/recombinants have been reported for many human fungal pathogens and some of the hybrid progenies have shown hybrid vigour and expanded clonally [95]. Along with the identified phylogenetic incompatibility and the existence of both *MAT* **a** and *MAT* **α** loci in several isolates, we speculate that mating among closely related strains (or in the same cluster) may have been common, localized, and contributed to adaptations and differentiations within and among geographical and ecological populations of *C. glabrata* in Qatar. Indeed, *C. glabrata* is able to mate, as shown in other studies [65]. As noted above, evidence for intermixing among clusters, and recombination within individual clusters were found within the Qatari population of *C. glabrata*. Specifically, high rates of phylogenetic incompatibility were found within five of the six clusters, with each consisting of genetically closely related isolates within Qatar. Closely related strains from across broad geographical scales have been reported in almost all human microbial pathogens, including human fungal pathogens. So far, most such clusters have been attributed to clonal expansions of specific genotypes. Indeed, our original hypothesis was that isolates from each of the six clusters of *C. glabrata* from Qatar were asexually related to each other due to recent clonal expansion, resembling the five genomics clades recognized in *C. auris* [86]. The high rates of phylogenetic incompatibility coupled with limited genetic variations within each of the clusters suggest that recombination probably occurred among closely related strains. Furthermore, given that several isolates have both *MAT*a and *MAT*α loci, the inferred recombination might at least partly involve progeny derived from the same meiosis of a parental strain, similar to inbreeding/selfing in homothallic fungi [96].

Among the 80 Qatari isolates, there was a broad range of MICs against each of the nine antifungal drugs. For isolates with available MICs against antifungal drugs with known resistance breakpoints for *C. glabrata*, nine were fluconazole resistant, 4four were resistant to caspofungin, and one was resistant to fluconazole, caspofungin, anidulafungin and micafungin. In this study, as per treatment guidelines, most patients infected with *C. glabrata* were treated with echinocandins. It is possible that the observed resistance to echinocandins emerged in response to treatment; however, we did not have evidence to confirm this phenomenon because only one isolate was sequenced per patient, and we did not have sequence data for isolates representing before and after drug therapy from these patients. Investigation of 103 antifungal and other virulence-related genes revealed that 22 of these candidate genes contain 31 missense mutations in strains with elevated MICs. Among those missense mutations, Asp554Gly in *PDR1* has been previously reported from fluconazole-resistant *C. glabrata* [32]. In addition, two genes, CAGL0K13024g and CAGL0K00110g, were downregulated by almost 6-fold in an azole-resistant strain compared to the azole-susceptible strain CBS138 during growth in medium containing 32 mg l^−1^ fluconazole [97]. However, none of the SNPs in these 22 genes cleanly separated the drug-resistant and drug-susceptible strains and the individual contributions of most of the missense mutations to antifungal susceptibility differences among the Qatari *C. glabrata* strains are likely to be small. Functional studies will be essential to validate the contribution of mutations on antifungal susceptibilities.

Interestingly, five missense mutations in *CDR2* and *PDR1* are prevalent in the 17 isolates with relatively high MICs. *CDR2* encodes efflux pumps, the development and overexpression of which have been considered as the most frequent mechanism for azole resistance in *Candida* species [98, 99]. Gain-of-function mutants of *PDR1*, as the major regulator of ABC transporters, have been demonstrated to result in azole resistance as well as gain of virulence in murine models of disseminated infection [100, 101]. Of note, the four mutations (Val91Ile, Leu98Ser, Ser76Pro and Thr143Pro) in *PDR1* have previously been described to occur in both azole-resistant and azole-susceptible strains [97, 102]. At least 12 different *PDR1* alleles recovered from azole-susceptible isolates contained combinations of eight different mutations and 58 different alleles seemed specific for azole-resistant isolates with 58 distinct mutations [101].

Additionally, GWAS analyses were conducted to identify potential antifungal-related variants at the genome scale. We performed GWAS analyses using FarmCPU to increase statistical power and reduce false positives in the presence of complex population structures or rare variants [76]. In addition, we applied a loose minor allele frequency threshold of 0.01, which prevents the removal of rare variants and included all identified variants. The GWAS analyses revealed several novel mutations associated with MIC variation among the strains. For instance, GWAS identified two missense mutations significantly associated with anidulafungin susceptibility (Fig. 4). One SNP resulted in an Asp442Ser mutation in the protein product of CAGL0A00913g, which was predicted to be involved in the cellular response to DNA damage [79]. The other missense mutation led to an Ala74Gly mutation in the protein product of *INO2*. The presence of a particular mutation in *INO2* may have a negative effect on the anidulafungin MICs. Given that *INO2* is an activator for the expression of *INO1* [80], and *INO1* was shown to be downregulated in a *C. glabrata* strain with a higher fluconazole MIC than a strain with a lower MIC [103], the missense mutation in *INO2* is hypothesized to have a negative impact on the binding ability of *INO2* to the promoter region of *INO1*. This, in turn, may lead to a reduction in the number of drug targets in *C. glabrata* strains, potentially increasing their tolerance to antifungal agents. Together, mutations in other genes and epigenetic mechanisms or interactions among multiple genes may play important roles in antifungal drug resistance in *C. glabrata*. Similar mechanisms for drug resistance have been demonstrated in several other fungi, including triazole resistance in *C. albicans* and amphotericin B resistance in *A. fumigatus* [104, 105].

We would like to point out that there are limitations to our GWAS analyses. First, the sample size of 80 strains was relatively small, which can influence the power to identify variants contributing quantitative differences in MICs among strains. Second, some mutations that influence antifungal susceptibility may go undetected if a stringent minor allele frequency threshold was applied in the preprocessing step, which may filter out such variants. In addition, as a number of novel variants were identified as associated with antifungal susceptibility differences, the mechanisms of their contributions remain unknown. To identify whether these mutations influence antifungal susceptibilities through the same or different pathways as those of known drug resistance mechanisms, further analyses using a combination of gene knockouts/knockdowns and/or transcriptomic, proteomic and metabolomic analyses, including those of membrane sterols are needed.

## Conclusions

Our study revealed that the clinical *C. glabrata* populations in Qatari consist of multiple divergent genetic clusters. Several clusters contained genetically highly similar isolates spanning several years in the same and/or different hospitals, indicative of nosocomial transmission within hospital settings or in the community. Intriguingly, five clusters of isolates showed robust evidence of phylogenetic incompatibility, suggesting that inbreeding/selfing probably played an important role in the evolution and divergence of these populations. Importantly, the Qatari *C. glabrata* population was genetically similar to the global collections, consistent with recent anthropogenic activities playing a significant role in the dispersal and gene flow of this species across many regions of the globe. Combined analyses of genome-wide SNPs and antifungal susceptibilities revealed several unknown mutations in genes that may be associated with antifungal drug resistance. However, the role of these mutations in conferring drug resistance would require validation using targeted gene swapping and/or site-directed mutagenesis approaches.

## Supplementary Data

Supplementary material 1

Supplementary material 2
