# Supplementary material for: Population genomic analyses reveal high diversity, recombination and nosocomial transmission among Candida glabrata (Nakaseomyces glabrata) isolates causing invasive infections
Source: Microb Genom. 2024 Jan 16;10(1):001179. doi: 10.1099/mgen.0.001179 (PMC10868614; doi:10.1099/mgen.0.001179)
Supplement: Supplementary material 1 [file mgen-10-1179-s001.pdf]

Population genomic analyses reveal high diversity, recombination, and nosocomial transmission among *Candida glabrata (Nakaseomyces glabrata)* isolates causing invasive infections

Yue Wang et al. 2023

Supplementary Materials

Supplementary Figure S1. Phylogeny showing *MSH2* SNP distribution among the 80 Qatari isolates. Presence of each SNP was marked using purple circle. Amino acid changes were labelled on the top.

Supplementary Figure S2. PCA of *C. glabrata* populations using high-resolution SNPs. Six major different genetic clusters were recognized with sporadic isolates.

Supplementary Figure S3. Π and Tajima's D distribution for different *C. glabrata* populations.

Supplementary Table S1: Metadata of the 80 Qatari samples included.

Supplementary Table S2: List of 103 antifungal and virulence related genes screened for mutations.

Supplementary Table S3. Pairwise SNP distance comparison among Qatar strains.

Supplementary Table S4. BLAST results of isolates with unidentified mating type.

Supplementary Table S5: SNPs of the 103 candidate genes present in the 80 Qatari *C. glabrata* sample.

Supplementary Table S6: GWAS results on flucytosine susceptibility in the Qatar population. Supplementary Table S7: GWAS results on anidulafungin susceptibility in the Qatar population.

Supplementary Table S8: SNPs that exclusively present in the 13 genetically divergent strains of *C. glabrata* in Qatar.

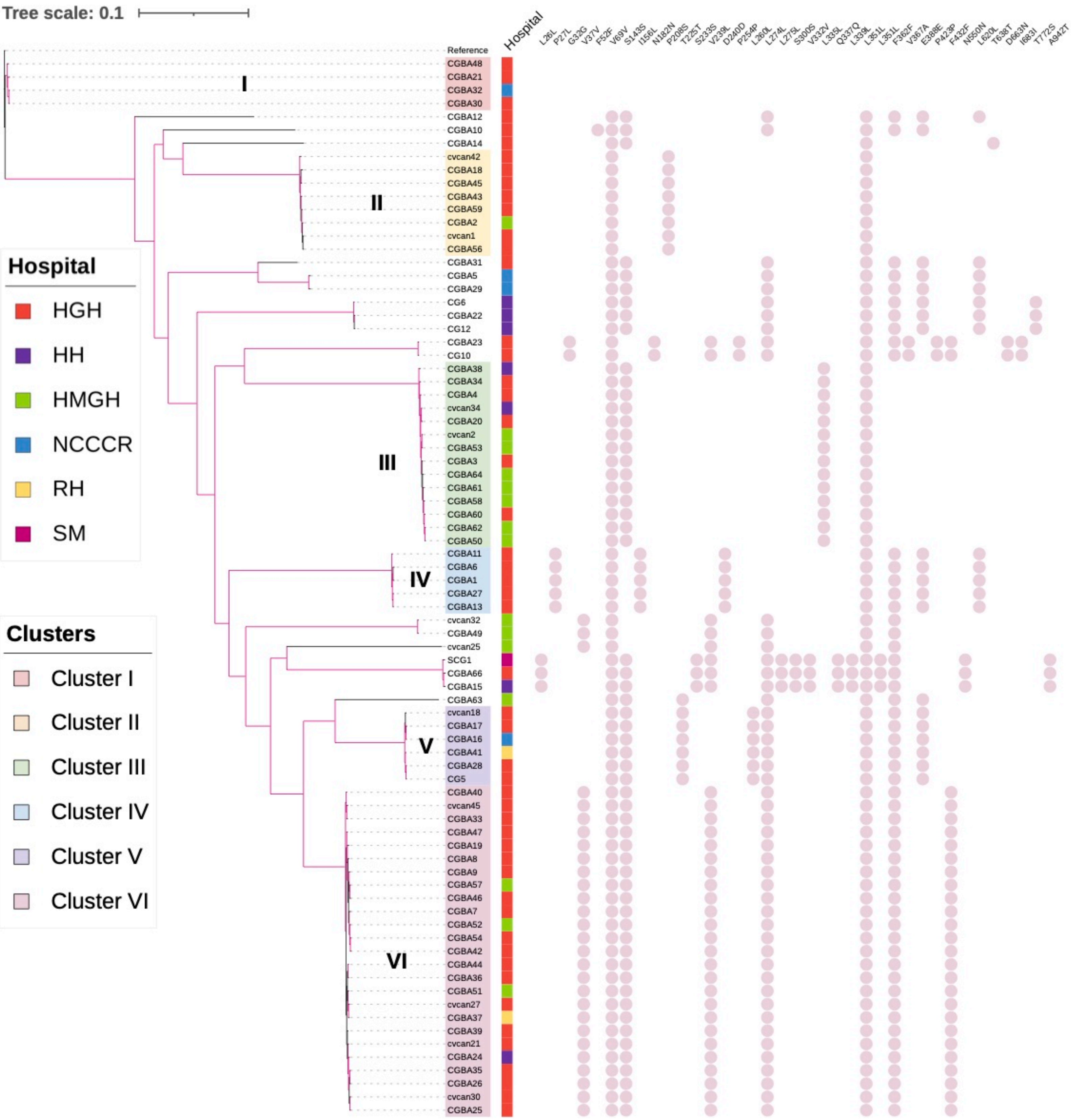

Tree scale: 0.1
Hospital
L26L
P27L
G32G
V37V
F52F
V69V
S143S
I156L
N182N
P208S
T225T
S233S
V239L
D240D
P254P
L260L
L274L
L275L
S300S
V332V
L335L
Q337Q
L339L
L351L
L351L
F362F
V367A
E388E
P423P
F432F
N550N
L620L
T638T
D663N
I683I
T772S
A942T
Reference
CGBA48
CGBA21
CGBA32
CGBA30
I
CGBA12
CGBA10
CGBA14
cvcan42
CGBA18
CGBA45
CGBA43
CGBA59
CGBA2
cvcan1
CGBA56
II
CGBA31
CGBA5
CGBA29
CG6
CGBA22
CG12
CGBA23
CG10
CGBA38
CGBA34
CGBA4
cvcan34
CGBA20
cvcan2
CGBA53
CGBA3
CGBA64
CGBA61
CGBA58
CGBA60
CGBA62
CGBA50
III
CGBA11
CGBA6
CGBA1
CGBA27
CGBA13
IV
cvcan32
CGBA49
cvcan25
SCG1
CGBA66
CGBA15
CGBA63
cvcan18
CGBA17
CGBA16
CGBA41
CGBA28
CG5
V
CGBA40
cvcan45
CGBA33
CGBA47
CGBA19
CGBA8
CGBA9
CGBA57
CGBA46
CGBA7
CGBA52
CGBA54
CGBA42
CGBA44
CGBA36
CGBA51
cvcan27
CGBA37
CGBA39
cvcan21
CGBA24
CGBA35
CGBA26
cvcan30
CGBA25
VI
Hospital
HGH
HH
HMGH
NCCCR
RH
SM
Clusters
Cluster I
Cluster II
Cluster III
Cluster IV
Cluster V
Cluster VI

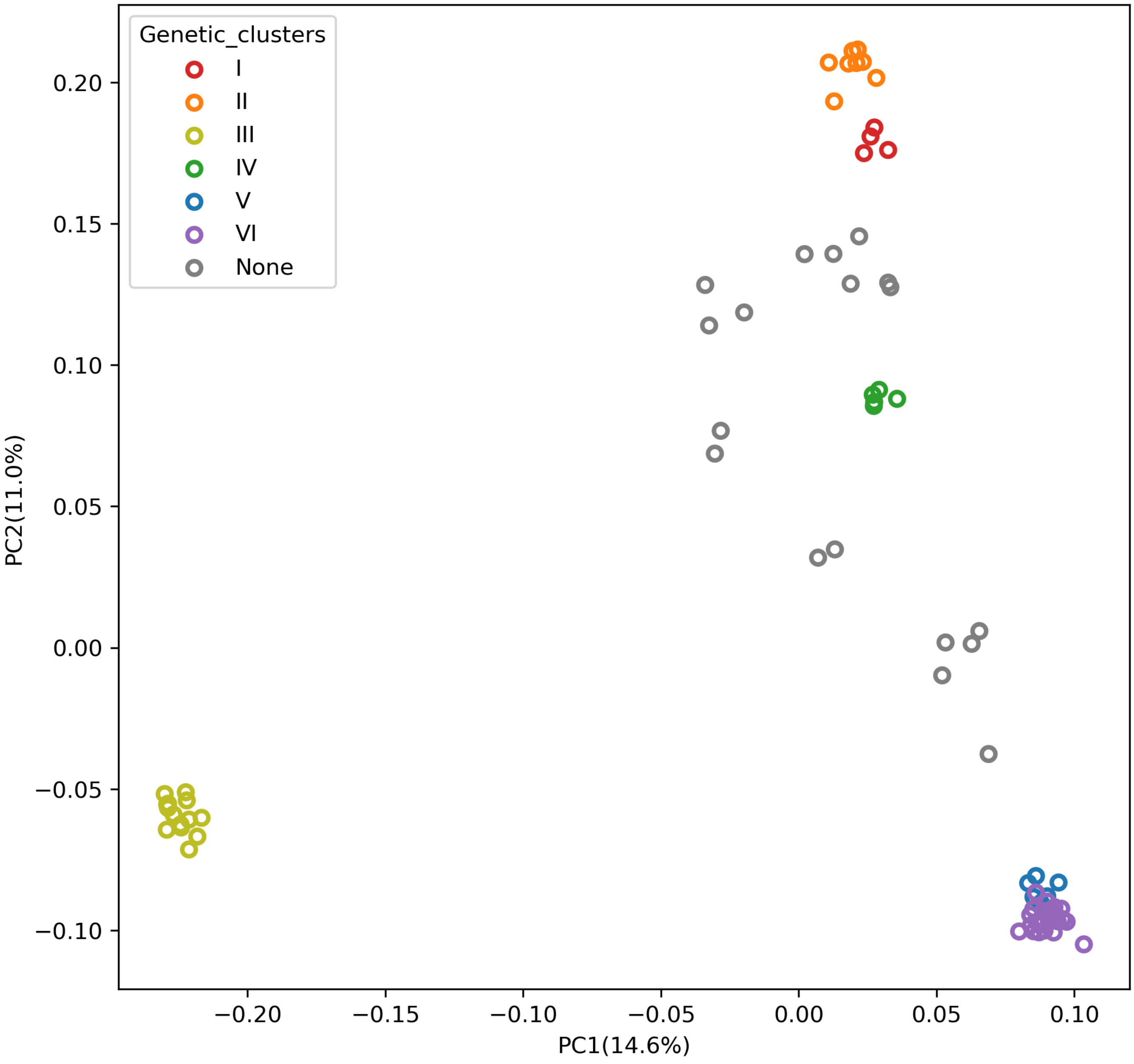
Genetic_clusters
I
II
III
IV
V
VI
None
0.20
0.15
0.10
0.05
0.00
−0.05
−0.10
PC2(11.0%)
−0.20
−0.15
−0.10
−0.05
0.00
0.05
0.10
PC1(14.6%)

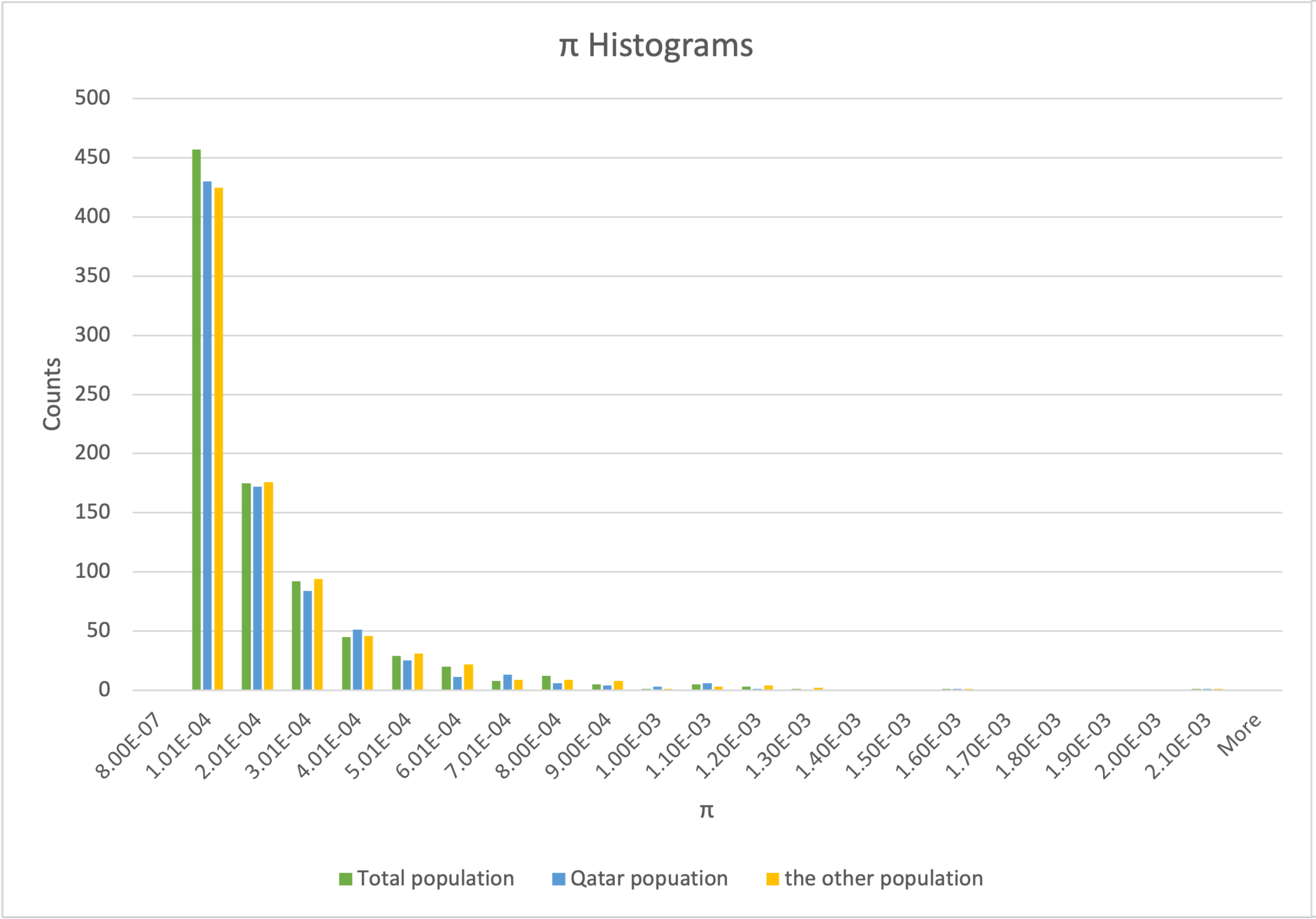
π Histograms
Counts
500
450
400
350
300
250
200
150
100
50
0
8.00E-07
1.01E-04
2.01E-04
3.01E-04
4.01E-04
5.01E-04
6.01E-04
7.01E-04
8.00E-04
9.00E-04
1.00E-03
1.10E-03
1.20E-03
1.30E-03
1.40E-03
1.50E-03
1.60E-03
1.70E-03
1.80E-03
1.90E-03
2.00E-03
2.10E-03
More
π
Total population
Qatar popuation
the other population

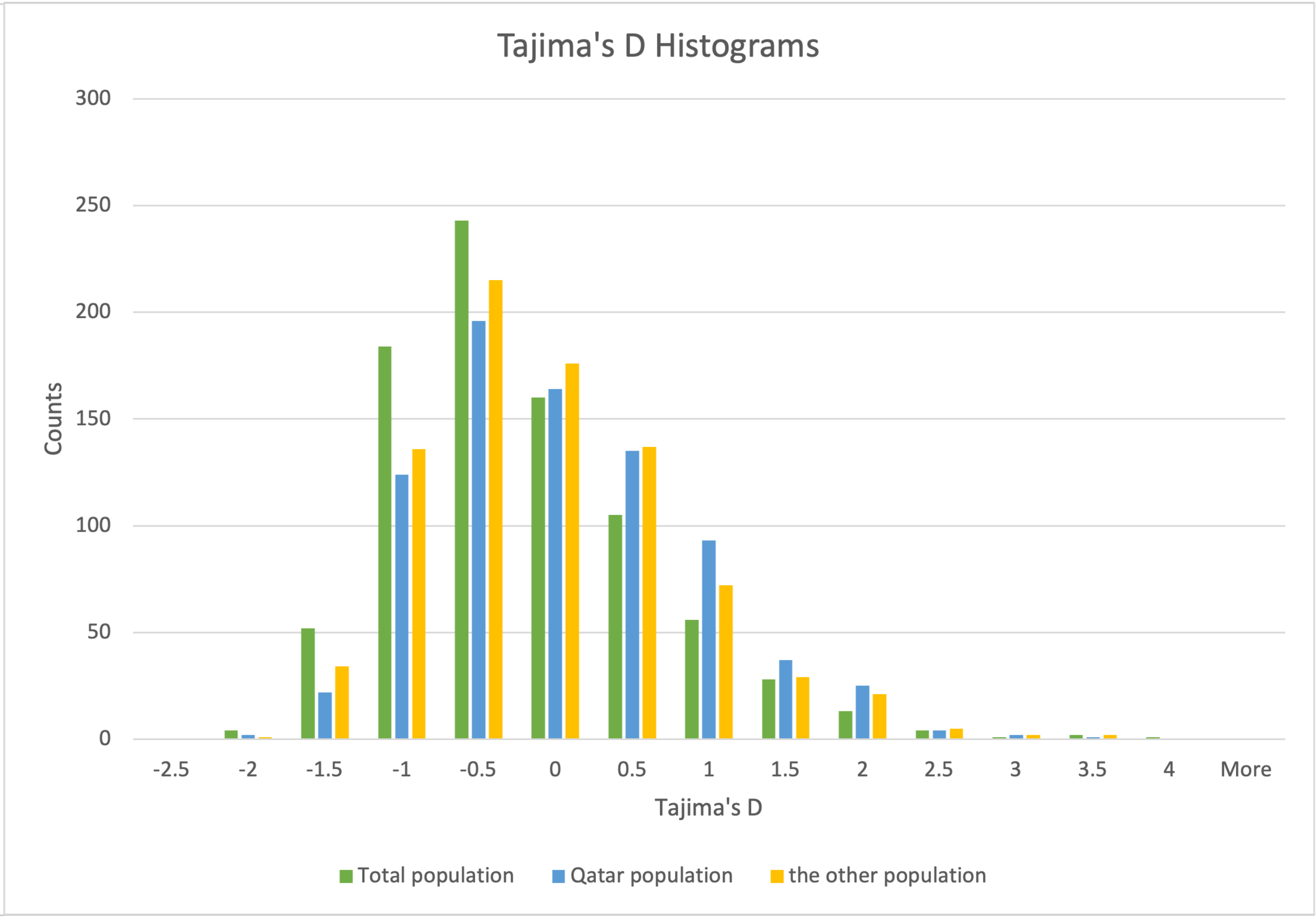
Tajima's D Histograms
Counts
300
250
200
150
100
50
0
-2.5
-2
-1.5
-1
-0.5
0
0.5
1
1.5
2
2.5
3
3.5
4
More
Tajima's D
Total population
Qatar population
the other population
